# Editorial: Understanding and Communicating Wildland Fire Smoke Risk

**DOI:** 10.3389/fpubh.2021.721823

**Published:** 2021-09-29

**Authors:** Gayle S. W. Hagler, Sarah B. Henderson, Sarah McCaffrey, Fay H. Johnston, Susan Stone, Ana Rappold, Wayne E. Cascio

**Affiliations:** ^1^United States Environmental Protection Agency, Office of Research and Development, Research Triangle Park, NC, United States; ^2^Environmental Health Services, British Columbia Centre for Disease Control (BCCDC), Vancouver, BC, Canada; ^3^Rocky Mountain Research Station, United States Forest Service, Fort Collins, CO, United States; ^4^Environmental Health Group, Menzies Institute for Medical Research, University of Tasmania, Hobart, TAS, Australia; ^5^Public Health Services, Department of Health, Tasmanian Government, Hobart, TAS, Australia; ^6^United States Environmental Protection Agency, Office of Air Quality Planning and Standards, Research Triangle Park, NC, United States

**Keywords:** wildland fire, air pollution, smoke, wildfire, PM2.5, risk communication

Globally, smoke from landscape fires—including wildland fire (encompassing wildfires and prescribed fires), agricultural burning, tropical deforestation fires, peat fires, and grass fires—is estimated to cause 339,000 deaths annually (1). Epidemiological, clinical, animal, and cellular studies support a positive association between short-term exposure to wildland fire smoke and premature death and respiratory disease, namely exacerbation of asthma, bronchitis, and pneumonia (2–4). Emerging data suggest that exposure to wildland fire smoke also increases the risk of clinical cardiovascular events, such as myocardial infarction, heart rhythm disturbances, stroke, out-of-hospital cardiac arrest, and acute heart failure (3, 5, 6). In addition, the potential for exposure to wildfire smoke during pregnancy to affect birth weight and prematurity is under exploration [(4, 7, 8) and references therein].

Quantifying the economic costs of wildfire smoke exposure, in the United States (US), the direct burden has been estimated to be more than $11 billion US dollars per year for short-term exposures and more than $76 billion per year for long-term exposures (9). Similar impacts have been estimated in Canada (10) and Australia (11). The indirect economic burden is likely much higher.

Wildfire smoke exposure risk is expected to increase in the future (12). Abatzoglou and Williams (13) determined that anthropogenic climate change and its effects on fuel aridity contributed to nearly a doubling of forest fire area in the western US over 1984–2015. On a global scale, scientists predict that climate change will result in “longer, hotter, and drier fire seasons” that increase the risk of severe wildfires and associated health impacts, including those from smoke exposure (14).

Given the increasing public health threat of wildland fire smoke, environmental health scientists are challenged to define at-risk populations and provide data and tools to support health protective information and decisions. Public health and healthcare professionals rely on these scientific advances to communicate about risk and risk reduction strategies. Predicting wildland fire smoke exposure is critical to risk reduction, and this must occur in concert with communication about health protective resources. For example, the severe acute respiratory syndrome coronavirus 2 (SARS-CoV-2) pandemic was a critical co-occurring public health risk during the 2020 and 2021 wildfire seasons (15, 16). Considering wildfire smoke and SARS-CoV-2 together, specialized guidance was required to communicate the interplay between these two risks (e.g., https://www.cdc.gov/coronavirus/2019-ncov/php/smoke-faq.html). Research and development of new technologies are needed to meet this complex and evolving challenge. This Research Topic brings together six publications providing important new data sets, end user tools, and insights into effective public health messaging and risk reduction actions.

## Smoke and Public Health Information Data and Tools

Assessing wildland fire smoke risk necessitates the fusing of information about smoke exposure and the affected population. To support a variety of potential smoke risk assessment investigations, Vargo combined the US Census Block Group Centers of Population with the National Oceanic and Atmospheric Administration (NOAA) Hazard Mapping System Smoke product covering a June 2010 through December 2019 time span. This resulting data set exceeding 59 million records can help accelerate research to understand smoke exposure in the United States and, in Vargo's words, “can be important for targeting funding, interventions, and communications to areas more often impacted by smoke from fires.” In Australia and Canada, two innovative end user tools have been developed to support public health response by combining critical data sets to inform timely action by individuals and public health agencies. The Air Quality Visualization (AQVx) tool, described by Williamson and Lucani, is a smoke risk management tool that combines modeled and measured PM_2.5_ (mass of particles with aerodynamic diameter <2.5 μm) concentrations, crowd-sourced smoke and symptom reports from the complementary AirRater smartphone application, fire information, population density, and locations of sensitive populations (aged care facilities, schools). A noted utility of AQVx is to support government messaging of risk to affected communities. The British Columbia Asthma Prediction System (BCAPS), described by Henderson et al. (https://maps.bccdc.ca/bcaps/), integrates smoke forecasts using an innovative methodology with health indicator data to predict the number of asthma inhaler dispensations over the coming days.

## Communicating Strategies to Reduce Smoke Exposure

Public health communication is most impactful if the delivery of information connects with the intended audience and motivates exposure reduction action. Research in the midst of an emergency is challenging to accomplish but provides critical insights. Marfori et al. conducted a qualitative study in Tasmania, Australia, immediately after a prolonged and significant wildfire smoke episode to elucidate the complexity of smoke risk communication. Interviewees who lived near to the fires were faced with concurrent property and smoke risks, and recommended actions were sometimes in conflict (e.g., work outside to reduce fuels around your home for fire prevention; stay inside and rest to reduce smoke exposure). Other interviewees, at a lower risk from the fire, wondered why the risks from the significant smoke exposure were not well-communicated by emergency services nor consistently covered by the media. In another study, Hano et al. described quantitative insights gained through volunteer participants in the US Environmental Protection Agency (EPA) Smoke Sense smartphone application and research study (https://www.epa.gov/air-research/smoke-sense-study-citizen-science-project-using-mobile-app). They found that the participants grouped by perspectives, not demographics, in terms of their responses to wildfire smoke risk.

To capture the current state of knowledge on risk reduction methods, Davison et al. hosted and summarized a web summit focused on the creation of cleaner indoor air spaces during smoke events. At the time this article was developed, it was noted that no national building guidelines existed. An important recent development was the release of the publicly available American Society of Heating, Refrigeration, and Air-conditioning Engineers (ASHRAE) Planning Framework for Protecting Commercial Building Occupants from Smoke During Wildfire Events (17). This precedes the full Guideline 44 document, expected in 2022.

Significant wildfire smoke episodes affect many regions worldwide and this risk is anticipated to grow in the future (18). On an international scale, sharing new technologies, data sets, and insights will support more effective risk communication strategies to protect public health.

## Author Contributions

GH and WC wrote the first draft of the manuscript. All authors contributed to manuscript revision, read, and approved the submitted version.

## Author Disclaimer

The summary thoughts presented here are those of the authors and should not be construed to represent any official U.S. Environmental Protection Agency, U.S. Department of Agriculture, or U.S. Government determination or policy.

## Conflict of Interest

The authors declare that the research was conducted in the absence of any commercial or financial relationships that could be construed as a potential conflict of interest.

## Publisher's Note

All claims expressed in this article are solely those of the authors and do not necessarily represent those of their affiliated organizations, or those of the publisher, the editors and the reviewers. Any product that may be evaluated in this article, or claim that may be made by its manufacturer, is not guaranteed or endorsed by the publisher.
